# Supplementary material for: Triangle-Mesh-Rasterization-Projection (TMRP): An Algorithm to Project a Point Cloud onto a Consistent, Dense and Accurate 2D Raster Image
Source: Sensors (Basel). 2023 Aug 8;23(16):7030. doi: 10.3390/s23167030 (PMC10458127; doi:10.3390/s23167030)
Supplement: Supplementary file 1 [file sensors-23-07030-s001.zip › sensors-2516022-supplementary.pdf]

Supplementary Materials: Triangle-Mesh-Rasterization-Projection (TMRP): An algorithm to project a point cloud into a dense accurate 2D raster image

1. Experiments #1—Process and mathematics

To obtain the source information (rx, ry), the point cloud $P_{Velo} = (X, Y, Z)$ must be transformed from the Cartesian coordinate system into spherical coordinates, see Eq. (1).

$$R = \sqrt{X^2 + Y^2 + Z^2}, \qquad \alpha = \frac{atan2(-Y, X)}{h_{res} \cdot (\pi/180°)}, \qquad \omega = \frac{atan2(Z, R)}{v_{res} \cdot (\pi/180°)} \tag{1}$$

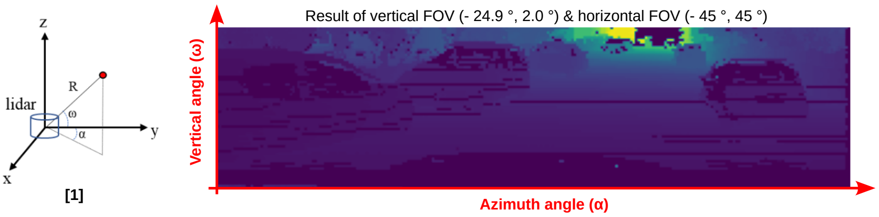

Figure S1. LiDAR skizze [1] *(left)*). 2D range image from 3D point cloud $P_{Velo} = (X, Y, Z)$ (of frame 20, `KITTI 2011_09_26_drive_0064` [2]).

2. Experiments #3—Process and mathematics

2.1. Overview

A 3D reconstruction of a three-dimensional object can be implemented using two cameras (cam_1, cam_3) and stereoscopic image processing algorithms (Figure S2b, orange colored pipeline). The quality of the predicted disparity map (output of deep stereo matching framework) and thus the point cloud depends strongly on the stereo matching method used. Deep stereo approaches have shown to be preferable to traditional methods in some areas[1] [3,4]. The big disadvantage of deep stereo matching frameworks (highly data-driven), is the need for training datasets. [5–7] Since there are no large benchmark datasets for industrial applications (Sec. 1 in main paper), we generate our own dataset. Our stereo dataset consists of lens undistorted and rectified image pairs (left and right) and associated ground truth disparity maps.

---

1 Despite decades of research, estimating the disparity of a stereo pair for uncooperative objects in the visible range is still an open problem[5]

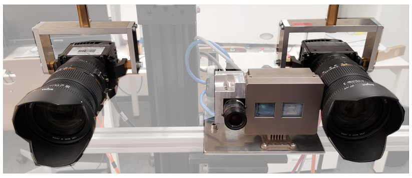

(a) Measurement setup: *(left-to-right)* right stereo camera cam_3, RGB camera (not used), time of flight (ToF) sensor $sensor_2$ (Basler blaze-101, as our reference system for ground truth disparities), left stereo camera cam_1.

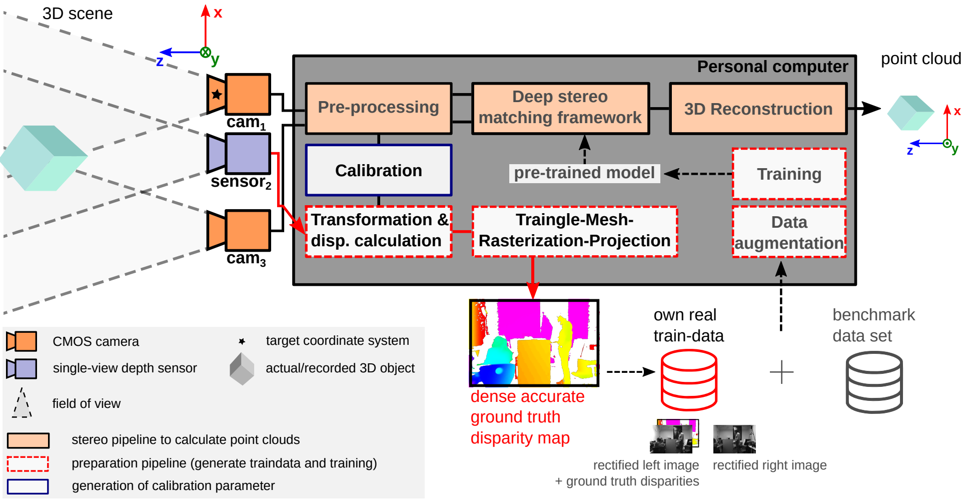

(b) Three-dimensional acquisition of a 3D object *(left)* via a passive stereo system consisting of cam_1, cam_3 and a stereo image processing pipeline (coloured orange). The quality of the generated point cloud *(right)* is strongly dependent on the stereo matching framework that predicts the disparity map. Due to the highly data-driven method of deep stereo, a training dataset is required. A stereo training dataset consists of left and right rectified images with corresponding ground truth disparity maps *(bottom-right)*, which should be as dense as possible. To create this, the system is expanded with a single-view depth sensor $sensor_2$. The processing chain for creating the ground truth disparity map is shown in red dashed boxes and includes our TMRP algorithm.

Figure S2. Application example for our TMRP algorithm: Create real stereo training dataset consisting of rectified left and right image and ground truth disparity map for deep stereo matching frameworks, e.g. FD-Fusion [8], AANet+ [9].

Figure S2a shows our measurement system. We use a passive stereo system (cam_1 and cam_3) and a time of flight (ToF) sensor $sensor_2$ (Basler blaze-101) as reference system for our (pseudo-real) ground truth disparity maps. The ToF sensor can also be replaced by any 3D sensor. Figure S2b shows the processing steps for generating a own training dataset. The six sub-steps are:

- **(1)** Camera **calibration** for estimating the calibration and rectification parameters for sub-step (3) (see Sec. 3 in main paper);
- **(2)** synchronous **data acquisition**;
- **(3.1) coordinate transformation**: see Eqs. (3) and (4);
  (3.2) disparity calculation $P(X, Y, d_x, rx, ry)$ according to Eq. (6).
- **(4) projection** of disparity points $P(X, Y, d_x, rx, ry)$ onto dense 2D target image using TMRP algorithm (Sec. 4 in main paper);
- **(5) data augmentation** [7] to increase dataset and thus increase process stability;
- **(6) training:** Creation of pre-trained model for deep stereo matching framework.

2.2. Calculate of (pseudo-real) disparities—mathematical description and definitions

2.2.1. Definition of the used parameters

Table S1 describes all relevant parameter for the calculations of ground-truth disparity maps. Eq. (2) shows the two projection matrices for a horizontal stereo camera arrangement. They include information like focal length f, horizontal base length T_x and optical centers (cx, cy) of cam_1 / cam_3 stereo camera.

Table S1. Relevant calibration and rectification parameter using *openCV*. The names of the cameras cam_1, $sensor_2$ (or $Ref3D$) and cam_3 refer to Figure S2b. The tuple of $\mathbf{R}_{cam1 \rightarrow Ref3D}$ and $\mathbf{T}_{cam1 \rightarrow Ref3D}$ performs a change of base from the coordinate system of the first camera *cam1* to the coordinate system of the second sensor $sensor_2$ resp. *Ref3D*. $\mathbf{R_{rect1}}$ performs a change of basis from the unrectified first camera's coordinate system to the rectified first camera's coordinate system (our world coordinte system, see Eqs. (3) and (4). $\mathbf{P_{rect1/3}}$ projects points given in the rectified camera coordinate system $cam1/cam3$ into the rectified camera's image cam_1/cam_3.

**Parameter**	**Definition**
$image\ size \in \mathbb{N}^2$	weight and height of image
$distCoeffs \in \mathbb{R}^5$	vector of distortion coefficients
$\mathbf{K1}, \mathbf{K2}, \mathbf{K3} \in \mathbb{R}^{3\times3}$	camera intrinsic matrix of $cam1$, $cam2$, $cam3$
$\mathbf{R}_{cam1 \rightarrow Ref3D} \in \mathbb{R}^{3\times3}$	rotation matrix
$\mathbf{t}_{cam1 \rightarrow Ref3D} \in \mathbb{R}^{1\times3}$	translation matrix
$\mathbf{R_{rect1}} \in \mathbb{R}^{3\times3}$	rotation matrix
$\mathbf{P_{rect1/3}} \in \mathbb{R}^{3\times4}$	projection matrix (of stereo camera system $cam1/cam3$)
$\mathbf{Q} \in \mathbb{R}^{4\times4}$	disparity-to-depth mapping matrix or reprojection matrix

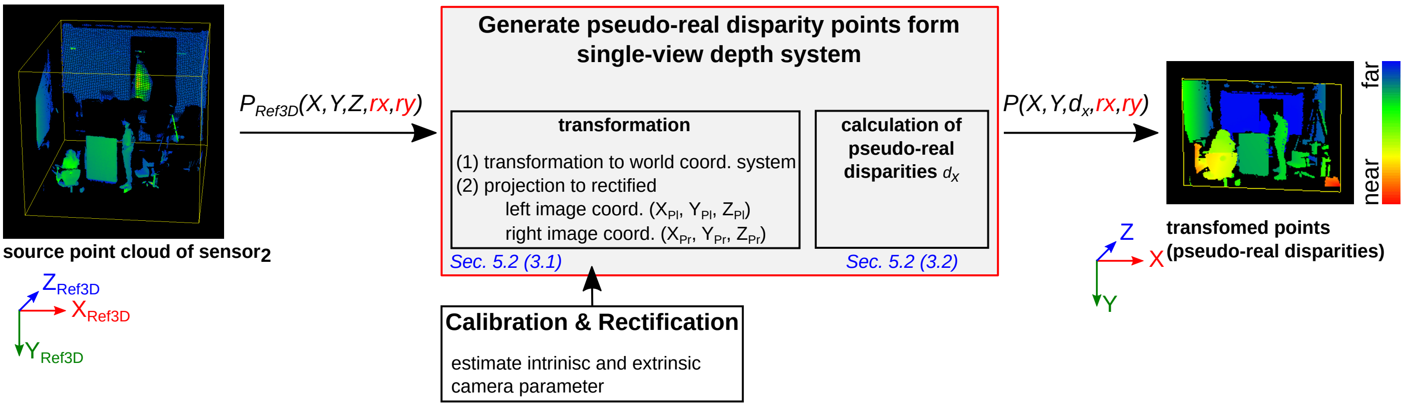

Figure S3. Sub-pipeline of block "transformation & disparity calculation" in Figure S2b. The captured raw point cloud from $sensor_2$ (single view depth sensor) *(left)* are transformed into the world coordinate system using the camera parameters from the calibration and rectification (Table S1). These transformed points are then projected into two point clouds (left and right raster) using the projection matrix $\mathbf{P_{rect1}}$ and $\mathbf{P_{rect3}}$ (Eqs. (2), (3) and (4)). The pseudo-real disparity points $P(X, Y, d, rx, ry)$ *(right)* are calculated according to Eq. (6).

$$\mathbf{P_{rect1}} = \begin{pmatrix} f & 0 & cx_1 & 0 \\ 0 & f & cy_1 & 0 \\ 0 & 0 & 1 & 0 \end{pmatrix}, \quad \mathbf{P_{rect3}} = \begin{pmatrix} f & 0 & cx_3 & T_x \cdot f \\ 0 & f & cy_1 & 0 \\ 0 & 0 & 1 & 0 \end{pmatrix} \tag{2}$$

2.2.2. Calculation of ground truth disparity points

Figure S3 shows the sub-pipeline "transformation (3.1) & disparity calculation (3.2)" of Figure S2b. Eq. (6) describes the calculation of the horizontal disparity values dx.

$$\begin{pmatrix} X_{\mathrm{Pl}} \\ Y_{\mathrm{Pl}} \\ Z_{\mathrm{Pl}} \end{pmatrix} \underset{\text{(left homogeneous)}}{=} \underbrace{\mathbf{P_{rect1}} \cdot \underbrace{\begin{bmatrix} \mathbf{R_{rect1}} & 0_{3\times1} \\ 0_{1\times3} & 1 \end{bmatrix} \cdot \underbrace{\begin{bmatrix} \mathbf{R}^{-1}_{Ref3D\rightarrow cam1} & -\mathbf{R}^{-1}_{Ref3D\rightarrow cam1}\mathbf{T}_{Ref3D\rightarrow cam1} \\ 0_{1\times3} & 1 \end{bmatrix} \cdot \begin{pmatrix} X_{Ref3D} \\ Y_{Ref3D} \\ Z_{Ref3D} \\ 1 \end{pmatrix}}_{\text{transformation to stereo coordinate } (cam_1)}}_{\text{transformation to world coordinate (rectified } cam_1)}}_{\text{transformation to world coordinate and projection to rectified left stereo image raster}} \tag{3}$$

$$\begin{pmatrix} X_{\mathrm{Pr}} \\ Y_{\mathrm{Pr}} \\ Z_{\mathrm{Pr}} \end{pmatrix} \underset{\text{(right homogeneous)}}{=} \underbrace{\mathbf{P_{rect3}} \cdot \underbrace{\begin{bmatrix} \mathbf{R_{rect1}} & 0_{3\times1} \\ 0_{1\times3} & 1 \end{bmatrix} \cdot \underbrace{\begin{bmatrix} \mathbf{R}^{-1}_{Ref3D\rightarrow cam1} & -\mathbf{R}^{-1}_{Ref3D\rightarrow cam1}\mathbf{T}_{Ref3D\rightarrow cam1} \\ 0_{1\times3} & 1 \end{bmatrix} \cdot \begin{pmatrix} X_{Ref3D} \\ Y_{Ref3D} \\ Z_{Ref3D} \\ 1 \end{pmatrix}}_{\text{transformation to stereo coordinate } (cam_1)}}_{\text{transformation to world coordinate (rectified } cam_1)}}_{\text{transformation to world coordinate and projection to rectified right stereo image raster}} \tag{4}$$

$$\begin{pmatrix} x_{\mathrm{Pl}} \\ y_{\mathrm{Pl}} \end{pmatrix} = \underbrace{\begin{pmatrix} X_{\mathrm{Pl}}/Z_{\mathrm{Pl}} \\ Y_{\mathrm{Pl}}/Z_{\mathrm{Pl}} \end{pmatrix}}_{\text{STEP 2.3}}, \qquad \begin{pmatrix} x_{\mathrm{Pr}} \\ y_{\mathrm{Pr}} \end{pmatrix} = \underbrace{\begin{pmatrix} X_{\mathrm{Pr}}/Z_{\mathrm{Pr}} \\ Y_{\mathrm{Pr}}/Z_{\mathrm{Pr}} \end{pmatrix}}_{\text{STEP 2.3}} \tag{5}$$

$$\begin{pmatrix} X \\ Y \\ d_x \\ rx \\ ry \end{pmatrix} \xleftarrow[\text{(result)}]{\text{pseudo-real ground truth}} \underbrace{d_x(X_{\mathrm{Pl}}, Y_{\mathrm{Pl}}) = \begin{cases} X_{\mathrm{Pl}}(Y_{\mathrm{Pl}}) - X_{\mathrm{Pr}}(Y_{\mathrm{Pl}}) & (d_x \geq 0) \\ NaN & (d_x < 0) \end{cases}, \quad \text{for} \quad (Y_{\mathrm{Pl}} == Y_{\mathrm{Pr}})}_{\text{calculate pseudo-real disparitie } d_x \text{ based on two point clouds (points}_{Pl} \text{ and points}_{Pr})} \tag{6}$$

References

1. You, J.; Kim, Y.K. Up-Sampling Method for Low-Resolution LiDAR Point Cloud to Enhance 3D Object Detection in an Autonomous Driving Environment. *Sensors* **2023**, *23*. https://doi.org/10.3390/s23010322.
2. Geiger, A.; Lenz, P.; Stiller, C.; Urtasun, R. Vision meets robotics: the KITTI dataset. *The International Journal of Robotics Research* **2013**, *32*, 1231–1237. https://doi.org/10.1177/0278364913491297.
3. Poggi, M.; Tosi, F.; Batsos, K.; Mordohai, P.; Mattoccia, S. On the Synergies between Machine Learning and Binocular Stereo for Depth Estimation from Images: a Survey, 2021, [arXiv:cs.CV/2004.08566].
4. Junger, C.; Notni, G. Optimisation of a stereo image analysis by densify the disparity map based on a deep learning stereo matching framework. In Proceedings of the Dimensional Optical Metrology and Inspection for Practical Applications XI. International Society for Optics and Photonics, SPIE, 2022, Vol. 12098, pp. 91 – 106. https://doi.org/10.1117/12.2620685.
5. Zama Ramirez, P.; Tosi, F.; Poggi, M.; Salti, S.; Di Stefano, L.; Mattoccia, S. Open Challenges in Deep Stereo: the Booster Dataset. In Proceedings of the Proceedings of the IEEE conference on computer vision and pattern recognition, 2022. CVPR.
6. He, J.; Zhou, E.; Sun, L.; Lei, F.; Liu, C.; Sun, W. Semi-synthesis: A fast way to produce effective datasets for stereo matching, 2021. https://doi.org/10.48550/ARXIV.2101.10811.
7. Rao, Z.; Dai, Y.; Shen, Z.; He, R. Rethinking Training Strategy in Stereo Matching. *IEEE Transactions on Neural Networks and Learning Systems* **2022**, pp. 1–14. https://doi.org/10.1109/TNNLS.2022.3146306.
8. Ferrera, M.; Boulch, A.; Moras, J. Fast Stereo Disparity Maps Refinement By Fusion of Data-Based And Model-Based Estimations. In Proceedings of the International Conference on 3D Vision (3DV), 2019.
9. Xu, H.; Zhang, J. AANet: Adaptive Aggregation Network for Efficient Stereo Matching. In Proceedings of the 2020 IEEE/CVF Conference on Computer Vision and Pattern Recognition, CVPR 2020, Seattle, WA, USA, June 13-19, 2020. IEEE, 2020, pp. 1956–1965. https://doi.org/10.1109/CVPR42600.2020.00203.
